# Supplementary material for: An enhanced pairing-free certificateless directed signature scheme
Source: PLoS One. 2022 Feb 17;17(2):e0263943. doi: 10.1371/journal.pone.0263943 (PMC8853546; doi:10.1371/journal.pone.0263943)
Supplement: S1 Table — (PDF) [file pone.0263943.s002.pdf]

**Notation**	**Description**	**Execution Ttime (ms)**
T_{SM}	Scalar multiplication on elliptic curve	0.165217
T_{Add}	point addition on elliptic curve	0.001404
T_P	bilinear pairing	4.441043
T_{MTP}	Map to point in G hash function	0.142682
T_H	Map to an element in Z_q^* hash function	0.001784
