# Supplementary figures and images for: An enhanced pairing-free certificateless directed signature scheme
Source: PLoS One. 2022 Feb 17;17(2):e0263943. doi: 10.1371/journal.pone.0263943 (PMC8853546; doi:10.1371/journal.pone.0263943)

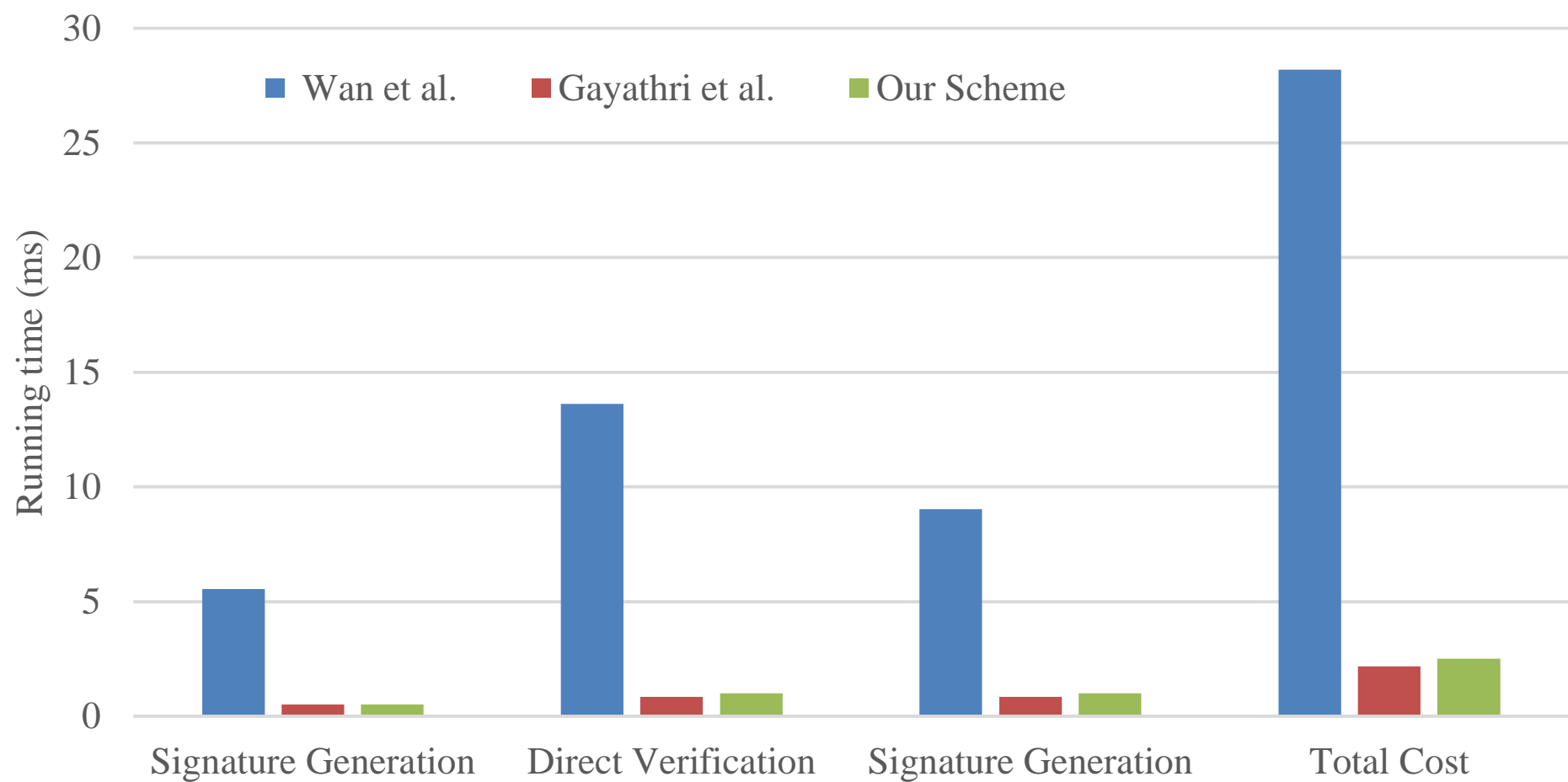

Supplement: S1 Fig — (PDF) [file pone.0263943.s003.pdf]

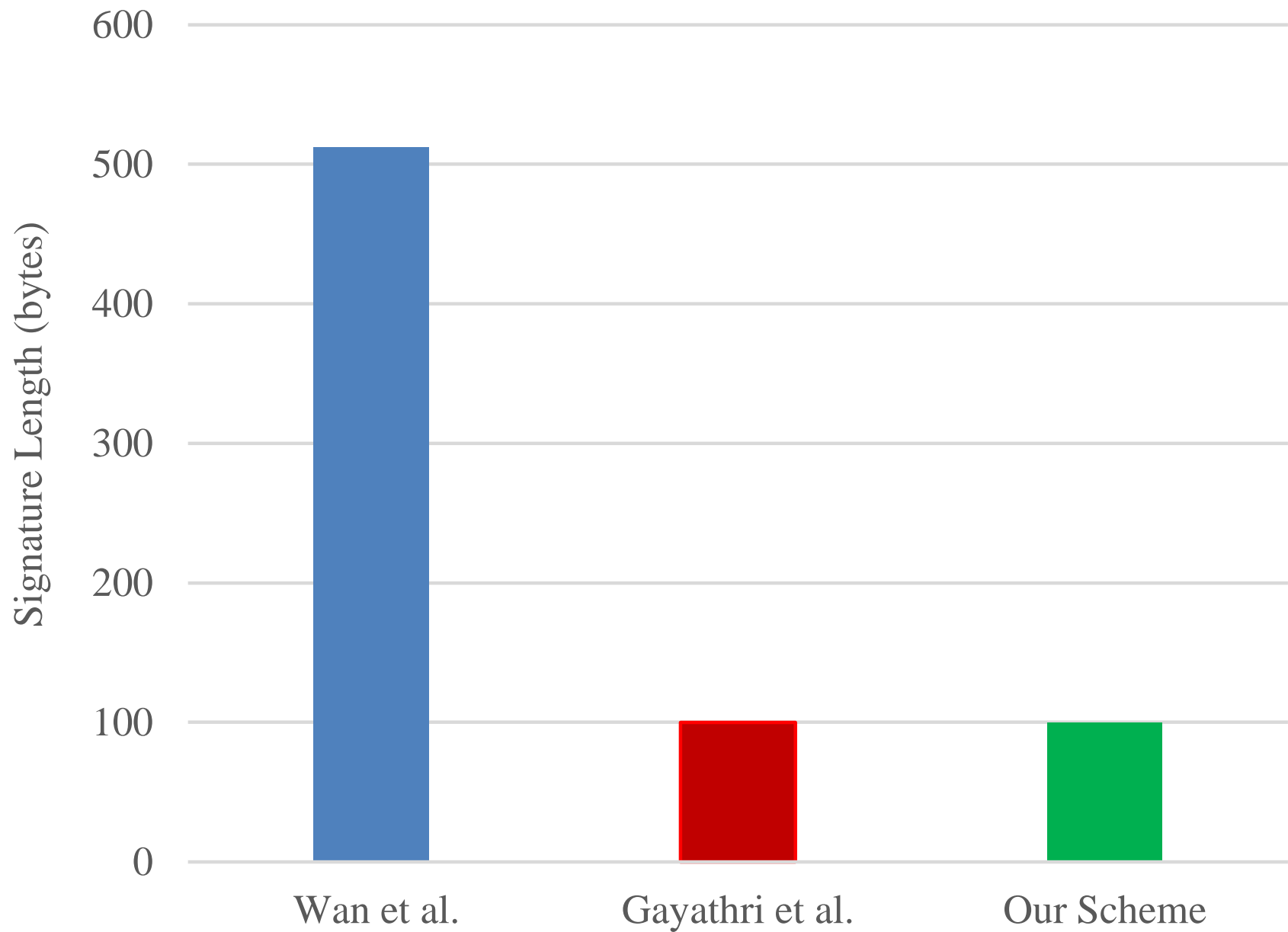

Supplement: S2 Fig — (PDF) [file pone.0263943.s004.pdf]
