# Supplementary material for: An enhanced pairing-free certificateless directed signature scheme
Source: PLoS One. 2022 Feb 17;17(2):e0263943. doi: 10.1371/journal.pone.0263943 (PMC8853546; doi:10.1371/journal.pone.0263943)
Supplement: Attachment — Submitted filename: Response to Reviewers.pdf [file pone.0263943.s005.pdf]

List of Responses

Dear Dr. Pandi Vijayakumar and Reviewers:

We quite appreciate your favorite consideration and the reviewer's insightful comments. Now we have revised our manuscript entitled "An enhanced pairing-free certificateless directed signature scheme" (ID: PONE-D-21-34945) exactly according to the reviewer's comments, and found these comments are very helpful for revising and improving our paper, as well as the important guiding significance to our researches. Revised portion are marked in red in the paper. We hope this revision can make our paper more acceptable. The main corrections in the paper and the responds to the reviewer's comments are as follows:

Responds to the reviewer's comments:

Reviewer #1:

Comments: The author is required to analyse the following security related papers in the introduction section. Moreover, the computational cost of the proposed work should be compared with the following papers.

1EAAP: Efficient anonymous authentication with conditional privacy-preserving scheme for vehicular ad hoc networks

Dual authentication and key management techniques for secure data transmission in vehicular ad hoc networks

An efficient anonymous mutual authentication technique for providing secure communication in mobile cloud computing for smart city applications

An efficient anonymous authentication and confidentiality preservation schemes for secure communications in wireless body area networks

EMBA: An efficient anonymous mutual and batch authentication schemes for vanets

BBAAS: Blockchain-Based Anonymous Authentication Scheme for Providing Secure Communication in VANETs

Response: We are very sorry for our negligence of the relevant works, which we have added in references [4-9]. Furthermore, we have reviewed the application of digital signature techniques in related schemes in the introduction section. In Section 5.3, we have added the analysis in terms of computational cost and communication performance of related schemes.

Reviewer #2: The author has given enough security analysis and also, the performance analysis suggests that, the work is novel and is suitable for publication in our journal. The following relevant works as citations in the proposed work to add value to its significance.

Efficient NTRU lattice-based certificateless signature scheme for medical cyber-physical systems

A practical group blind signature scheme for privacy protection in smart grid

Efficient and secure anonymous authentication with location privacy for IoT-based WBANs

Response: We are very sorry for our negligence of the relevant works, which we have already added in references [10-12]. In addition, we have described the application of these schemes in the introduction section.

We tried our best to improve the manuscript and made some changes in the manuscript. These changes will not influence the content and framework of the paper.

Thank you very much for Dr. Pandi Vijayakumar and Reviewers' warm work earnestly, and hope that the correction will meet with approval.

Best regards!
Yours sincerely,
Kaiqin Yang
